# Double Narrowband Induced Perfect Absorption Photonic Sensor Based on Graphene–Dielectric–Gold Hybrid Metamaterial

**DOI:** 10.1186/s11671-022-03724-1

**Published:** 2022-09-04

**Authors:** Zhimin Liu, Shanshan Zhuo, Fengqi Zhou, Xiao Zhang, Yipeng Qin, Xin Luo, Cheng Ji, Guangxin Yang

**Affiliations:** School of Science, East China Jiaotong University, Nanchang, 330013 China

**Keywords:** Plasmon-induced absorption, Graphene, Metasurface, Sensor

## Abstract

Double narrowband induced perfect absorption in the terahertz region is achieved in a graphene–dielectric–gold hybrid metamaterial, whose physical mechanism is analyzed using the coupled-mode theory (CMT), which agreed well with the finite-difference time-domain (FDTD) simulation. This study found that the Fermi level of graphene can be adjusted to improve the absorptivity when the refractive index (RI) **n**_**d**_ of the chosen dielectric cannot achieve a good absorption effect. In addition, the blue shift of absorption spectrum can be used in the design of dual-frequency electro-optical switches, of which the modulation degree of amplitude (MDA) can reach as high as 94.05% and 93.41%, indicating that this is a very promising electro-optical switch. Most significantly, the RI sensing performance is investigated, which shows an ultra-high absorption sensitivity *S*_A_ = 4.4°/RIU, wavelength sensitivity *S*_λ_ = 9.8°/RIU, and phase shift sensitivity *S*_φ_ = 2691°/RIU. At last, an interesting finding is that the two peaks (R1 and R2) of plasmon-induced absorption (PIA) show different polarization characteristics (insensitive or sensitive) to the incident light angle; this polarization-sensitive is particularly important for the PIT/PIA-based optical polarizers. Undoubtedly, this paper is of great significance to the research and design of terahertz photonic devices and sensors.

## Introduction

Surface plasmon polaritons (SPPs) [1, 2] have been extensively studied in the micro-nano optics. SPPs, as carriers of information and energy transmission, are originated from the interaction between photons and electrons on the surface of metal or insulator. SPPs are confined to and transported along the metal medium interfaces so that the surface structure of metal or metal-like medium can be changed to control the transmission of SPPs [3]. Thus, SPPs provide new methods for electromagnetic wave transmission. SPPs can break the limit of diffraction, and control photons and device miniaturization in the sub-wavelength band, indicating that SPPs have good prospects for nano-integrated optical chip applications [4]. Recently, graphene, as a new material with a single layer of two-dimensional honeycomb lattice structure [5], displays a metal-like property in the terahertz band. And scientists have confirmed through experiments that graphene can also excite the SPPs in specific wavebands [6]. Compared to metal-based SPPs, graphene-based SPPs have many unparalleled advantages and optical properties [7–9]. First of all, dynamic tunability is an outstanding advantage of grapheme [10]; we indirectly control the conductivity and dielectric constant of graphene by applying gate voltage to regulate the Fermi level, thus achieving dynamic transport modulation of SPPs. Secondly, the field localization and enhancement effects of graphene-based SPPs are more remarkable than metal-based SPPs [8]. And the propagation range can be from near-infrared to terahertz frequency. In addition, strong dispersion is a significant advantage of graphene-based SPPs, the group index can reach more than 1000 [11], which far exceeds metal-based SPPs. Accordingly, some plasmon devices based on graphene-based SPPs have been widely applied to various fields such as optical switches [12], optical absorbers [13–15], sensing technology [16, 17], plasmonic enhancement [18, 19] and others. The most typical case is the plasmon-induced transparency (PIT) [20–23], which is produced by the destructive interference between resonant SPPs modes. Compares to the electromagnetically induced transparency (EIT) [24] obtained by the destructive quantum interference, experimental conditions of PIT are lower than EIT. According to the previous researches, various photonic devices based on PIT have been proposed, including photoelectric switches [25, 26], absorbers [27, 28], slow light devices [29], nano-imaging, modulators [30], etc.

For most PIT systems, perfect absorption has always been a challenge because the absorption rates of most PIT systems only reach about 50% [31]. In order to solve the problem of low absorption based on PIT, the idea of induced reflection in Tamm plasmon systems is proposed [32, 33], based on which some researchers designed a plasmon-induced reflection (PIR) [34] system, which achieved significant absorption effect. The concept to PIR is the opposite of PIT and rarely reported in graphene-based SPPs devices. And another concept of PIR is plasmon-induced absorption (PIA) [27, 35, 36], where the resonance peaks and dips of PIR and PIA can all correspond to the PIT. Moreover, the designed system is usually composed of metal and medium, among which the metal substrate is mainly designed to reflect all incident light to make sure the transmission rate is 0. The absorption rate is significantly enhanced compared with some PIT systems. Thus, it is meaningful to design metamaterials by combining patterned graphene with dielectric and metal materials to realize perfect absorption, PIA, and other related characteristics. Accordingly, this study proposes a graphene–dielectric–gold hybrid metamaterial structure to achieve perfect PIA in the terahertz band.

In this paper, a single-layer graphene patterned structure based on a graphene–dielectric–gold hybrid metamaterial is put forward to achieve the double narrowband induced perfect absorption. Firstly, the Fermi level of graphene is adjusted to improve the absorptivity when the refractive index (RI) **n**_***d***_ of the chosen dielectric cannot achieve good absorption effect. Secondly, the results of the FDTD simulation agree well with the CMT numerical simulation; and the blue shift phenomenon is applied to design an electro-optical switch. Most significantly, its RI sensing performance is investigated, which shows an ultra-high absorption sensitivity *S*_A_ = 4.4°/RIU, wavelength sensitivity *S*_λ_ = 9.8°/RIU, and phase shift sensitivity *S*_φ_ = 2691°/RIU, indicating an extraordinary sensor. In addition, we find the two peaks (R1 and R2) of PIA show different polarization characteristics (insensitive or sensitive) to the incident light angle. And this polarization-sensitive is particularly important for the PIT/PIA-based optical polarizers. To summarize, the proposed structure provides many potential advantages. On the one hand, the provided structure has multiple means of modulation, enabling the structure to be multifunctional. On the other hand, the patterned graphene structure based on a graphene–dielectric–gold substrate is simple and monolayer, which is easy to manufacture. Therefore, the proposed structure provides a new way to realize terahertz photonic devices and sensors.

## Methods

**Fig. 1:**
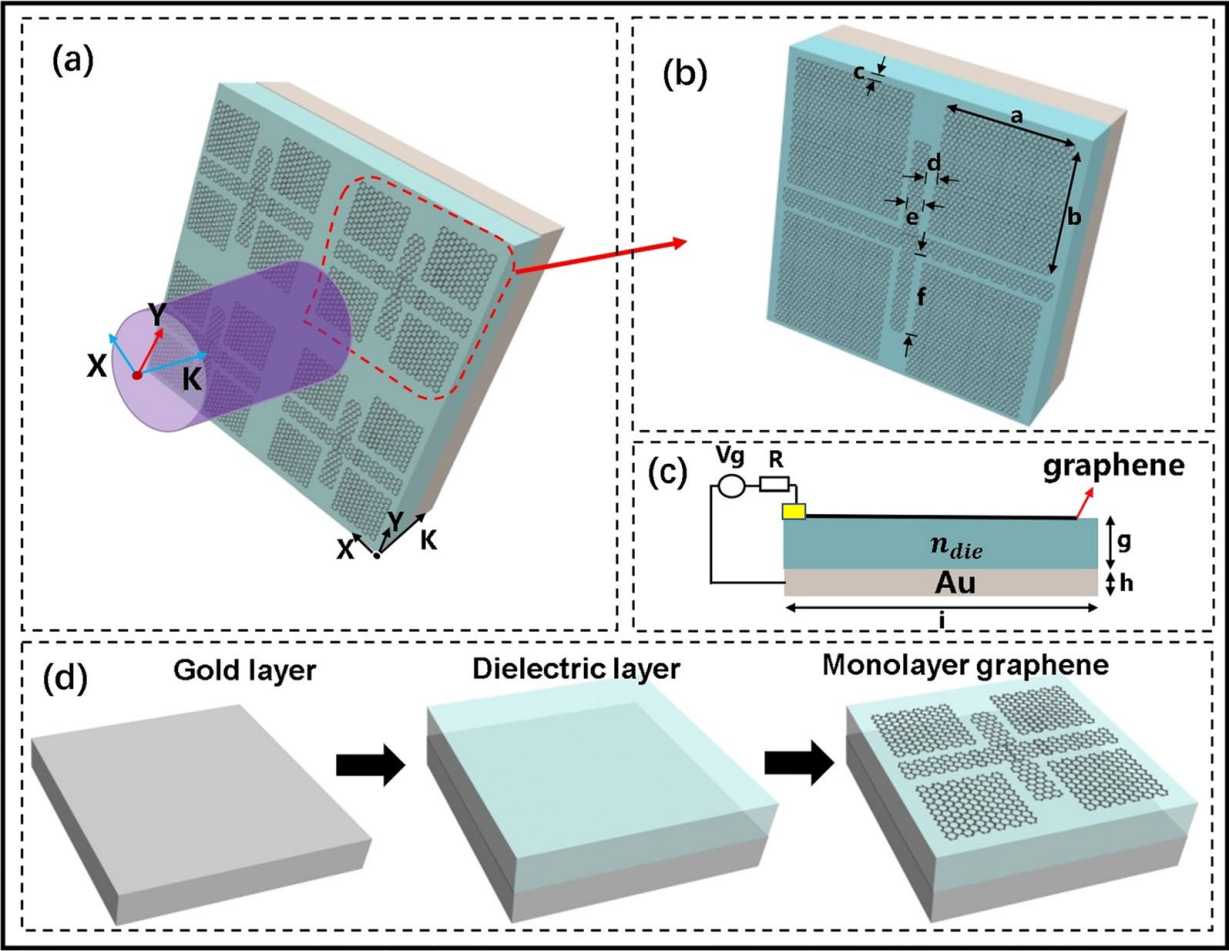
**a** Patterned structure of periodic monolayer graphene. **b** Single-layer graphene patterned unit structure. **c** The cross-sectional view of the structure and circuit diagram. **d** Preparation diagram of the provided structure metamaterial. Here, the geometric parameters of the structure are as follows: *a* =* b* = 1.5 μm, *c* = *d* = 0.2 μm, *e* = 0.3 μm, *f* = 1.4 μm, *i* = 4 μm; *g* = 1.6 μm, *h* = 0.5 μm

A graphene–dielectric–gold hybrid metamaterial is designed to achieve the PIR and perfect PIA in the terahertz band, as shown in Fig. 1a, the substrate of the proposed structure is composed of a metal layer and a dielectric layer, with a single-layer graphene placed above. And the unit structure of the periodic structure is shown in Fig. 1b; the patterned graphene consists of four graphene blocks (FGBs) and a cross-shaped graphene (CSG). Figure 1d indicates the preparation process of the provided structural metamaterial. Then, Fig. 1c exhibits the cross-sectional view of the proposed structure, the bottom layer is a gold layer, which is mainly used for reflecting most of the light; the upper layer is a dielectric layer; and the single-layer graphene metamaterial is set on the dielectric layer. The patterned graphene is connected to the electrode, and we can apply a gate voltage ***v***_**g**_ (see the circuit diagram in Fig. 1c) to adjust the Fermi level of graphene. The formula is as follows [37]:

1 $$E_{f} = \hbar \nu_{f} \sqrt {\frac{{\pi \varepsilon_{0} \varepsilon_{d} v_{g} }}{{ed_{0} }}} ,$$

where *v*_*f*_ and *d*_0_ refer to the Fermi velocity and the thickness of the dielectric layer, respectively.

This work is based on the Lumerical FDTD Solutions software for simulation calculations. Here, both the X-axes and *Y*-axes directions are set as the periodic boundary conditions while the *Z*-axes direction is the perfect matched layers. Meanwhile, the incident light in the *x*-polarization direction is applied to the surface of the provided structure. In addition, according to the random phase approximation (RPA) theory, the surface optical conductivity ***σ***_**g**_ of graphene can be given [38]:

2 $$\sigma_{{\text{g}}} = \sigma^{{\text{intra}}} + \sigma^{{{\text{inter}}}} ,$$

3 $$\sigma^{{\text{intra}}} = \frac{{2ie^{2} k_{{\text{B}}} T}}{{\pi \hbar^{2} (\omega + i\tau^{ - 1} )}}In\left[ {2\cosh \left( {\frac{{E_{f} }}{{2k_{{\text{B}}} T}}} \right)} \right],$$

4 $$\sigma^{{\text{inter}}} = \frac{{ie^{2} (\omega + i\tau^{ - 1} )}}{{4\pi k_{{\text{B}}} T}}\int_{0}^{ + \infty } {\frac{G(\xi )}{{\hbar^{2} (\omega + i\tau^{ - 1} )^{2} /(2k_{{\text{B}}} T)^{2} - \xi^{2} }}} {\text{d}}\xi ,$$

where *τ* and *T* are the carrier relaxation time and the temperature (*T* = 300 K), respectively. *G*(*ξ*) = sinh(*ξ*)/[cosh(*E*_*f*_ /*k*_B_*T*) + cosh(*ξ*)] and *ξ* = *ε*/*k*_B_*T* with *k*_B_ being the Boltzmann constant. Due to *k*_B_*T* ≪ *E*_*f*_ in the terahertz band, *σ*^inter^ can be ignored. Thus, the conductivity of graphene can be expressed by:

5 $$\sigma_{{\text{g}}} = \frac{{ie^{2} E_{f} }}{{\pi \hbar^{2} (\omega + i\tau^{ - 1} )}}.$$

**Fig. 2:**
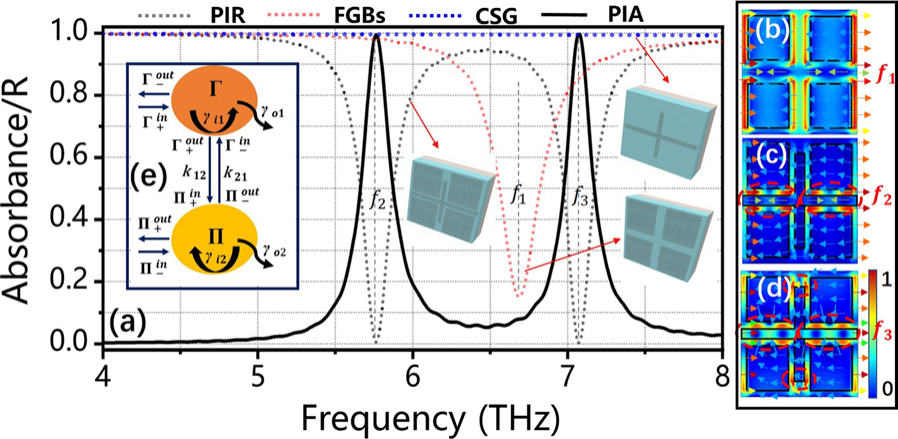
**a** Reflection and absorption spectrum of the PIR and PIA at E_*f*_ = 1.0 eV. **b**–**d** The electric field distribution at *f*_1_ = 6.684 THz, *f*_2_ = 5.758 THz and *f*_3_ = 7.064 THz, respectively. **e** The theoretical coupling between resonant modes

Firstly, when the FGBs of the structure are exposed to the linear polarized light, an obvious reflection valley (red dotted curve) is generated at *f*_1_ = 6.684 THz in Fig. 2a, we call it bright mode; when the CSG is irradiated by the linear polarized light, we get the blue dotted curve as shown in Fig. 2a, i.e., we call it dark mode. The PIR effect is generated when the FGBs are combined with CSG and the linear polarized light is vertical. Concretely, the two reflection valleys correspond to two resonance frequencies (*f*_2_ = 5.758 THz, *f*_3_ = 7.064 THz). Here, the black solid curve expresses the absorption spectrum of the proposed structure in Fig. 2a, which we call PIA effect. Moreover, the physical mechanisms of the PIR effect and PIA effect were discussed by the electric field around the proposed structure, as shown in Fig. 2b–d. Among them, Fig. 2b is the electric field diagram of FGBs at *f*_1_ = 6.684 THz, which shows the electric field energy is distributed at the edge of FGBs, indicating that the resonance reflection valley is excited by the edge of FGBs; additionally, Fig. 2c, d shows the electric field diagrams of two resonance reflection valleys of the PIR. In particular, the first resonance valley at *f*_2_ = 5.758 THz (see Fig. 2c) shows that the electric field energy is mainly distributed between FGBs and CSG, indicating that the first resonance valley is mainly contributed by the interaction between FGBs and CSG; the second resonance valley at *f*_3_ = 7.064 THz (see Fig. 2d) expresses the same distribution as well, besides, there is energy around the upper edge of the CSG, showing the second resonance valley is mainly affected by CSG. Here, the refractive index RI of the dielectric layer, the mobility of graphene and the Fermi level of graphene are **n**_**d**_ = 2, ***μ*** = 1.5 m^2^/(Vs) and ***E***_***f***_ = 1.0 eV, respectively.

Secondly, the physical mechanisms of the PIR effect and PIA effect can also be analyzed by the CMT, as shown in Fig. 2e. Here, Г and П represent the complex amplitudes of the two modes, respectively; Г_±_^in/out^ and П_±_^in/out^ correspond to the input and output waves of the two modes, respectively. In addition, the subscript “±” and “in/out” denote the same/opposite direction and the entering/exiting of the incident light. The coupling relationship between the two modes is as follows [39, 40]:

6 $$\left( {\begin{array}{*{20}c} {\gamma_{\Gamma } } & { - {\text{i}}\mu_{\Gamma \Pi } } \\ { - {\text{i}}\mu_{\Pi \Gamma } } & {\gamma_{\Pi } } \\ \end{array} } \right).\left( \begin{gathered} b_{\Gamma } \hfill \\ b_{\Pi } \hfill \\ \end{gathered} \right) = \left( {\begin{array}{*{20}c} { - \gamma_{o\Gamma }^{ - 1/2} } & 0 \\ 0 & { - \gamma_{o\Pi }^{ - 1/2} } \\ \end{array} } \right).\left( \begin{gathered} \Gamma_{ + }^{in} + \Gamma_{ - }^{in} \hfill \\ \Pi_{ + }^{in} + \Pi_{ - }^{in} \hfill \\ \end{gathered} \right),$$

where *μ*_ПГ_ and *μ*_ПГ_ represent the coupling coefficients of the two modes, respectively; *γ*_Г(П)_ = (*iω* − *iω* − *γ*_*i* Г(П)_  − *γ*_*o* Г(П)_), where *γ*_*i* Г(П)_ and *γ*_*o* Г(П)_ are the inter-loss coefficient and the extra-loss coefficient, respectively. According to the law of energy conservation, the coupling relationship between the two antennas can be expressed as follows:

7a $$\Gamma_{ + }^{{{\text{in}}}} = {\text{retain}},$$

7b $$\Gamma_{ - }^{{{\text{in}}}} = \Pi_{ - }^{{{\text{out}}}} e^{i\varphi } ,\Pi_{ + }^{{{\text{in}}}} = \Gamma_{ + }^{{{\text{out}}}} e^{i\varphi } ,$$

7c $$\Gamma_{ \pm }^{{{\text{out}}}} = \Gamma_{ \pm }^{{{\text{in}}}} - b_{\Gamma } \cdot \gamma_{o\Gamma }^{ - 1/2} ,\Pi_{ \pm }^{out} = \Pi_{ \pm }^{in} - b_{\Pi } \cdot \gamma_{o\Pi }^{ - 1/2} ,$$

where *φ* = Re(*β*)·*h*_1_ represents the phase difference between the two resonant modes П and Г. There is a gold layer at the bottom of the structure to reflect all the incident light, so the energy passing through the structure is 0. Therefore, entering the second resonant mode satisfies: П_−_^in^ = П_+_^out^e^*i*2*ψ*^, where *ψ* = Re(*β*)·*h*_2_ represents the phase difference between mode П and the metal layer. Therefore, the transmission and reflection coefficients are as follows:

8 $$t = \frac{{\Pi_{ + }^{{{\text{out}}}} }}{{\Gamma_{ + }^{{{\text{in}}}} }} = e^{i1\varphi } - \xi_{1} \cdot \gamma_{o\Gamma }^{ - 1/2} e^{i1\varphi } - \xi_{2} \cdot \gamma_{o\Pi }^{ - 1/2} ,$$

9 $$r = \frac{{\Gamma_{ - }^{{{\text{out}}}} }}{{\Gamma_{ + }^{{{\text{in}}}} }} = e^{i2\varphi } e^{i2\psi } - \xi_{1} \cdot (\gamma_{o\Gamma }^{ - 1/2} e^{i2\varphi } e^{i2\psi } + \gamma_{o\Gamma }^{ - 1/2} ) - \xi_{2} \cdot (\gamma_{o\Pi }^{ - 1/2} e^{i2\varphi } e^{i2\psi } + \gamma_{o\Pi }^{ - 1/2} e^{i1\varphi } ),$$

among them:

10 $$\xi_{1} = \frac{{N_{1} M_{2} - X_{1} N_{2} }}{{X_{2} M_{1} - X_{1} X_{2} }},\;\xi_{2} = \frac{{N_{1} X_{2} - M_{1} N_{2} }}{{M_{2} M_{1} - X_{1} X_{2} }},$$

11a $$M_{1} = \gamma_{\Gamma } - \gamma_{o\Gamma }^{ - 1} e^{i2\varphi } e^{i2\psi } ,\;M_{2} = - \gamma_{\Pi } + \gamma_{o\Pi }^{ - 1} e^{i2\psi } ,$$

11b $$N_{1} = \gamma_{o\Gamma }^{ - 1/2} + \gamma_{o\Gamma }^{ - 1/2} e^{i2\varphi } e^{i2\psi } ,\;N_{2} = \gamma_{o\Pi }^{ - 1/2} e^{i1\varphi } + \gamma_{o\Pi }^{ - 1/2} e^{i1\varphi } e^{i2\psi } ,$$

11c $$X_{1} = i\mu_{\Gamma \Pi } + \gamma_{o\Gamma }^{ - 1/2} \gamma_{o\Pi }^{ - 1/2} e^{i1\varphi } e^{i2\psi } + \gamma_{o\Gamma }^{ - 1/2} \gamma_{o\Pi }^{ - 1/2} e^{i1\varphi } ,$$

11d $$X_{2} = i\mu_{\Pi \Gamma } + \gamma_{o\Gamma }^{ - 1/2} \gamma_{o\Pi }^{ - 1/2} e^{i1\varphi } e^{i2\psi } + \gamma_{o\Gamma }^{ - 1/2} \gamma_{o\Pi }^{ - 1/2} e^{i1\varphi } ,$$

therefore, the transmission and reflection coefficients of the structure are: *T* = |*t*|^2^, *R* = |*r*|^2^, respectively; to sum up, the absorption rate of the structure: *A* = 1 − *T* − *R*.

## Results and Discussion

**Fig. 3:**
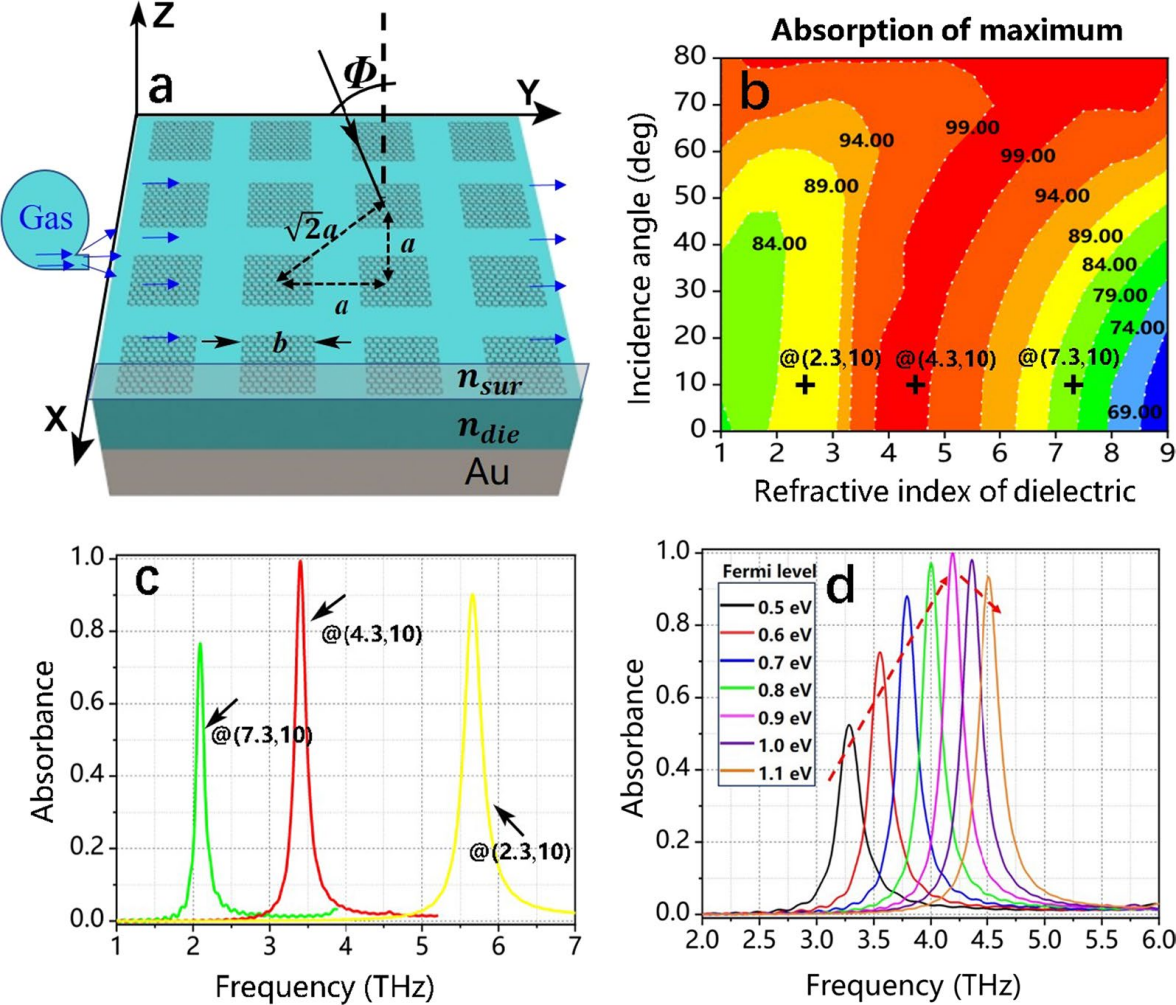
**a** The periodic structure of four graphene blocks. **b** Three-dimensional diagram of the maximum absorption with incident light *Φ* and refractive index *n*_d_ of the dielectric. **c** The absorption spectrum at positions @(2.3, 10), @(4.3, 10) and @(7.3, 10) of (**b**), respectively. **d** The FDTD numerical absorption spectrum with the Fermi level of graphene changing from 0.5 to 1.1 eV (*n* = 2)

In order to obtain the optimal absorption effect and parameters, we made a detailed investigation on the bright mode mentioned above first, and the structure diagram is shown in Fig. 3a, where ***Φ*** expresses the incident light angle. FGBs are placed above the dielectric layer and the gold layer **A**_**u**_; moreover, since the RI ***n***_***sur***_ of the surrounding dielectric can be affected by the change of gas concentration, a gas simulator is designed above the structure, which the detected gas can pass through. As shown in Fig. 3b, the absorption results vary with the incident angle ***Φ*** and the **RI** ***n***_***d***_ of the medium, and the contours represent different absorption values. What’s more, Fig. 3c shows the absorption diagrams at positions **@**(2.3, 10), **@**(4.3, 10) and **@**(7.3, 10) of Fig. 3b, respectively. Here, the X- and Y-values of **@**(X, Y) denote the RI ***n***_***d***_ of the medium and the incident angle ***Φ***, respectively. Among them, the absorption value at position **@**(4.3, 10) is almost 100%, which indicates the proposed structure can achieve perfect absorption. The FDTD numerical absorption spectrum with the Fermi level of graphene changing from 0.5 to 1.1 eV is shown in Fig. 3d. And the gate voltage *v*_g_ of graphene varies from 1.15 to 2.53 V, which has been experimentally proven that the range is feasible. With *E*_*f*_ being increased from 0.5 to 0.9 eV, the maximum absorption gradually increases and reaches 100%, then decreases during 0.9–1.1 eV. This phenomenon can be explained by the effective impedance [41] *Z* = (((1 + *S*_11_)^2^ − *S*_21_^2^)/((1 − *S*_11_)^2^ − *S*_21_^2^))^1/2^ where |*S*_11_| = |*r*|^2^ = *R* and |*S*_21_| = |*t*|^2^ = *T*. In Fig. 3d, we can discover the graphene meets excellent impedance matching (*Z* = 1) at *E*_*f*_ = 0.9 eV. However, the impedance does not match well when *E*_*f*_ ≠ 0.9 eV and the reflection appears so that the absorption decreases. Therefore, the Fermi level of graphene can be changed to improve the absorption when that of the selected dielectric is not ideal.

**Fig. 4:**
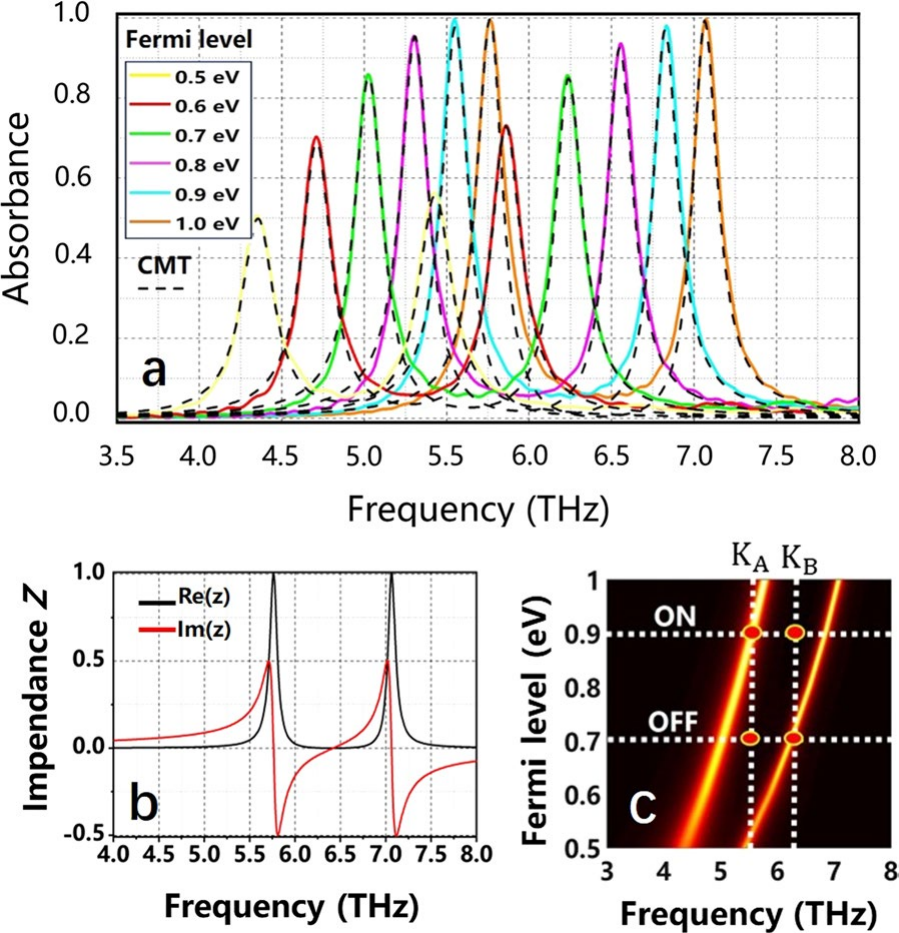
**a** The spectra of the FDTD numerical and CMT simulation with the Fermi level changing from 0.5 to 1.0 eV. **b** The real and imaginary parts of the impedance *Z* when *E*_*f*_ = 1.0 eV. **c** Three-dimensional diagram of absorption with the Fermi level and frequency. Here, the discussed structure is Fig. 1a and other parameters are *n*_d_ = 2, *n*_sur_ = 1

According to the previous section, we can adjust the parameters of the structure to improve its absorptivity when the FGBs are combined with CSG and the linear x-polarized light is vertical. Therefore, we chose the RI ***n***_**d**_** = 2, *n***_**sur**_** = 1** of the dielectric layer and the surrounding dielectric, respectively, and adjust the Fermi level to improve double absorption peaks in this section. The FDTD numerical absorption spectrum and the CMT fitting results with the Fermi level increasing from 0.5 to 1.0 eV are shown in Fig. 4a. Here, the solid lines represent the results of the FDTD numerical and the dotted lines for the CMT results. The FDTD results fit well with the CMT numerical simulation. We can see that the two absorption peaks gradually increase to almost 100% with the Fermi level changing from 0.5 to 1.0 eV. This is due to the fact that the impedances of both absorption peaks are perfectly matched when *E*_*f*_ = 1.0 eV. The real and imaginary parts of the impedance *Z* are exhibited in Fig. 4b, and it reveals that the real impedances of two resonance frequencies (*f*_2_ = 5.758 THz, *f*_3_ = 7.064 THz) are 1, manifesting that the two peaks reach 100% absorption.

Figure 4(c) displays the three-dimensional evolution diagram of absorption with Fermi level and frequency, there is an obvious blue shift phenomenon, which can be applied to the design of dual-frequency electro-optical switches in practical applications. As shown in Fig. 4c, at ***K***_**A**_, the absorption amplitude *K*_ON_ at 5.543 THz is 100%, the electro-optical switch is set as “ON” state with the Fermi level being 0.9 eV; while the absorption amplitude *K*_OFF_ is 5.95% at *E*_*f*_ = 0.7 eV, corresponding to the “OFF’’ state of the switch. Thus, the modulation degree of amplitude (MDA) at ***K***_**A**_ is 94.05%. Here, the MDA can be calculated by MDA = (*K*_ON_ − *K*_OFF_)/*K*_ON_ × 100% [42]. Similarly, the MDA at ***K***_**B**_ is 93.41% (*f*_*B*_ = 6.233 THz). Since the single-layer graphene structure is easier to realize under the experimental conditions, some electro-optic switches based on single-layer patterned graphene are shown in Table 1, which clearly illustrate the excellent performance of the proposed switch. The MDA of the two frequencies can reach as high as 94.05%, 93.41%, respectively. In addition, the absorption of PIT can only reach about 50%, whereas that of PIA can reach as high as 100%. Consequently, the proposed structure is of great significance in both electro-optical switches and solar energy absorption devices.

**Table 1:** Comparison of switches based on single-layer graphene

	Modulation mode	MDA (%)	Material structure	PIT/PIA
[43]	Three resonance points	80.01, 61.37, 50.97	Single-layer graphene	EIT
[12]	Four resonance points	77.70, 58.90, 75.40, 77.60	Single-layer grapheme	PIT
[44]	One resonance points	84.20	Single-layer graphene	PIT
[21]	Four resonance points	90.10, 80.10, 94.50, 84.70	Single-layer grapheme	PIT
This paper	Two resonance points	94.05, 93.41	Single-layer graphene	PIA

**Fig. 5:**
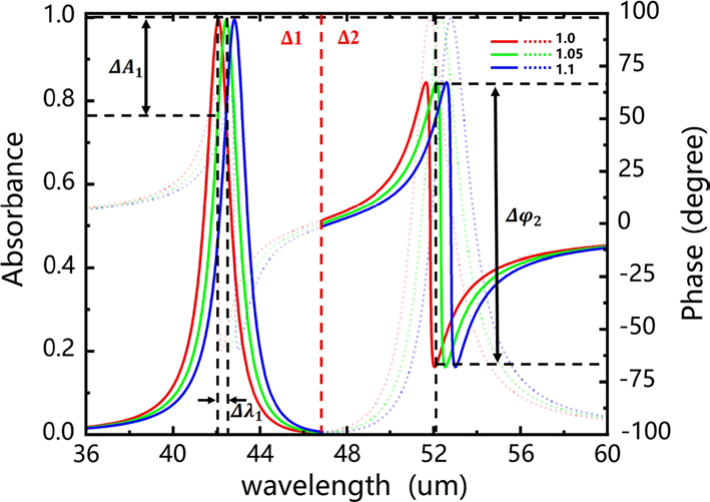
Absorption spectrum and the phase shift diagram when the refractive index *n*_sur_ is increased from 1 to 1.1 by a step of 0.05. Here, the discussed structure is Fig. 1a and other parameters are *n*_d_ = 2, *n*_sur_ = 1

In recent years, most of the work based on SPP sensing has been realized by detecting the SPP wave transmission at the interface between air and metal [45]. The gas concentration on the metal surface is changed to regulate the refractive index (RI) of the metal surface. In this section, we directly adjust the RI ***n***_***sur***_ of the surrounding dielectric of the whole structure to discuss the sensing performance with setting ***E***_***f***_ = 1.0 eV and ***n***_**d**_** = 2**. As shown in Fig. 5, the CMT fitting absorption spectrum and the phase shift diagram are exhibited when the RI ***n***_**sur**_ of the surrounding dielectric is increased from 1 to 1.1 by a step of 0.05. Here, we divide the two absorption peaks into two parts, **Δ1** and **Δ2,** with a red dotted line for clear analysis. The subscript of the obtained parameter is set as 1 for part **Δ1**, and 2 for part **Δ2**. When the RI ***n***_**sur**_ is adjusted from 1.0 to 1.05 (**Δ*n***_**d**_** = **0.05), the parameters are shown in Table 2.

**Table 2:** Parameters of the absorption, wavelength, phase shift

	Absorption	Wavelength	Phase shift
Δ1	0.2182	0.3723	121.17
Δ2	0.2214	0.4927	134.57
**S** of this paper	4.4	9.8	2691
**S** of [46]	3.8	3.0	2560

Here, **Δ**1(2) = **|*X***_*n*=1_ − ***X***_*n*_ _=_ _1.05_**|** (*X* = *A*, *λ* and *φ*) represents the changes of absorption amplitude, wavelength and phase shift, respectively; **S**_x_ = **max[Δ**1, **Δ**2]/**Δ*n***_**d**_ represents the sensitivities of absorption **S**_**A**_, wavelength **S**_***λ***_ and phase shift **S**_***φ***_, respectively. Compared with Ref. [46], it can be seen that the phase shift sensitivity of the proposed structure is significantly improved from 2560°/RIU to 2691°/RIU. Therefore, it shows a wide application prospect in sensors.

**Fig. 6:**
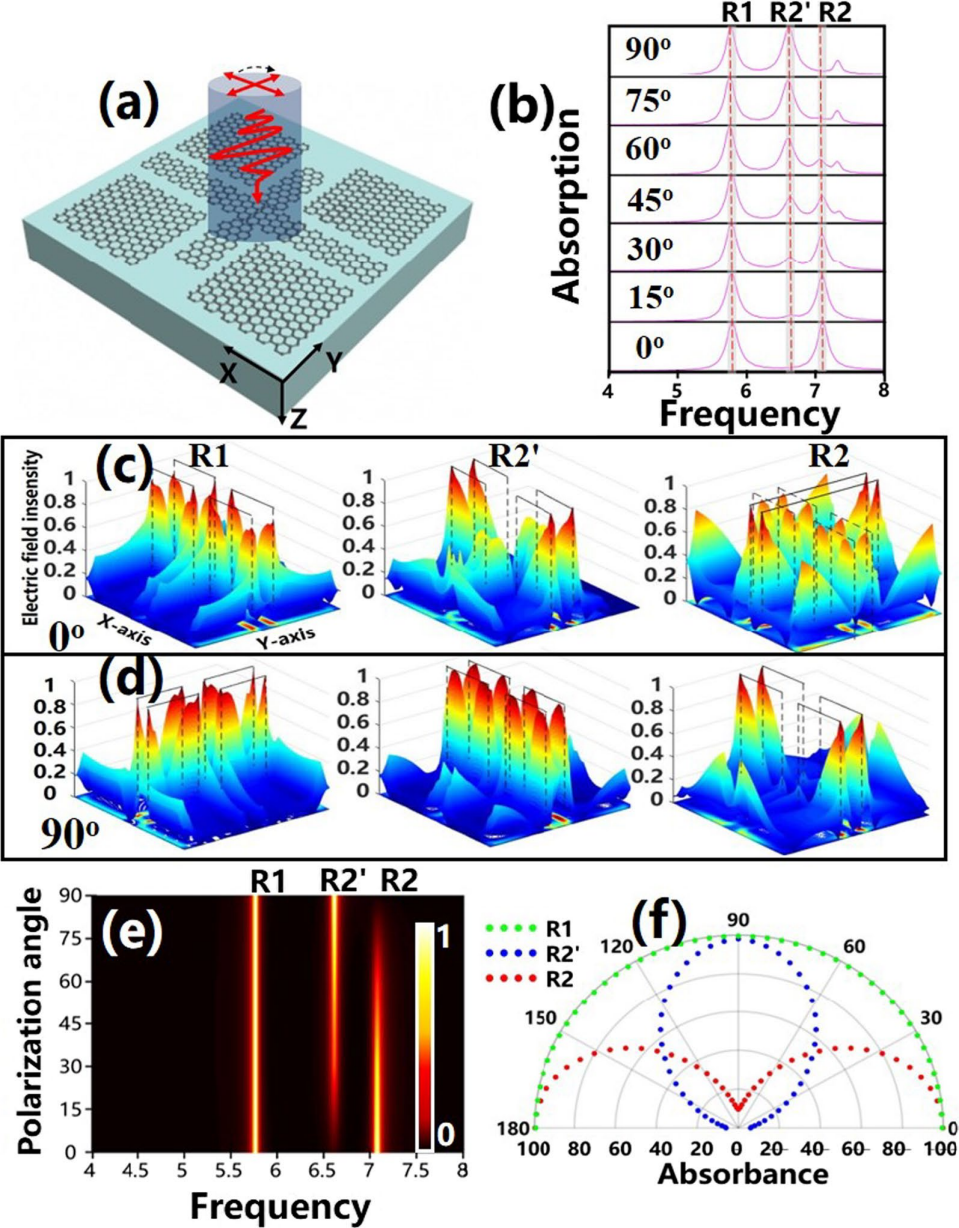
**a** Single-layer graphene patterned unit structure. **b** Absorption spectrum with polarization angle varying from 0° to 90°. **c**, **d** The electric field around the structure at *f*_R1_, *f*_R2'_ and *f*_R2_ with polarization angles being 0° and 90°, respectively. **e** Three-dimensional diagram of absorption with polarization angle and frequency. **f** Polar coordinates diagram variation of the polarization angle from 0° to 180°

The polarization angle of the incident light of the proposed structure in Fig. 6a is further investigated in this section. As shown in Fig. 6b, the two absorption peaks show different polarization characteristics when the polarization angle is changed from 0° (*x*-polarization) to 90° (*y*-polarization) by a step of 15°. In general, peak R1 shows sensitive characteristics while R2 shows insensitive characteristics to the incident light angle. Specifically, the absorptivity of R1 remains unchanged at *f*_**R1**_ = 5.758 THz, while that of R2 decreases gradually from 100 to 15% at *f*_R2_ = 7.064 THz; and a new peak R2' is formed at *f*_R2'_ = 6.373 THz, whose absorptivity can reach almost 100%. Thus, we consider that R1 and R2 are mainly controlled by FGBs and CSG, respectively; and R2 is moved to R2' due to the asymmetry of CSG. The physical mechanism of this phenomenon is analyzed by the electric field around the proposed structure in Fig. 6c , d to confirm the above analyses. When the polarization angle is changed from 0° to 90°, we find that most electric field energy is always distributed around FGBs at *f*_**R1**_, manifesting that the formation of resonance peak R1 is mainly excited by FGBs; at *f*_R2_, most energy is transferred from the Y-axis to the X-axis edge of CSG as the polarization angle is adjusted from 0° to 90°, indicating that R2 is mainly affected by CSG. Therefore, the above analyses are in line with our view that R1 is polarization-insensitive due to the symmetry of FGB, while R2 and R2' are polarization-sensitive due to the asymmetry of CSG. The three-dimensional evolution of absorption with polarization angle and frequency is plotted in Fig. 6e. It’s clear that there is no change in peak R1 with the polarization angle transforming from 0° to 90°; while peak R2 increases gradually; peak R2' decreases gradually, which can more vividly describe the change process of the two resonance peaks. Additionally, the polar coordinates of resonant dips generated under varied polarization angles from 0° to 180° are plotted in Fig. 6f, where the radius of the polar coordinates is the absorption amplitude, showing the evolution of the absorption resonance peaks. As the polarization angle gradually increases, the peak R1 shows a semicircle shape (green dotted line), indicating that the peak R1 does not vary with the polarization angle. However, the two peaks R2 and R2′ show opposite changes (red and blue dotted lines). These two variations are caused by the symmetry and asymmetry of FGBs and CSG, respectively. In summary, the provided structure has good polarization characteristics and has a good prospect for polarizers and other applications.

## Conclusion

To conclude, this paper provides a double narrowband induced perfect absorption. Firstly, the bright mode is investigated to enhance the absorption by adjusting the Fermi level. Then, the corresponding characteristics of PIA are discussed by the CMT numerical and the FDTD simulation, and the FDTD simulation is in good agreement with the CMT numerical results. In addition, we design an electro-optical switch based on PIA, and the MDA can reach as high as 94.05% and 93.41%. Noteworthy, the proposed structure is applied to sensors, which exhibited an ultra-high absorption sensitivity **S**_**A**_ = 4.4°/RIU, wavelength sensitivity **S**_**λ**_ = 9.8°/RIU and phase shift sensitivity **S**_**φ**_ = 2691°/RIU. In the end, with the polarization angle varying from 0° to 90°, we further find the absorption peak R1 is polarization-insensitive, while both R2 and R2' are polarization-sensitive. For the above analysis, the provided structure shows multiple means of modulation, enabling the structure to be multifunctional. Therefore, this designed structure will make sense in terahertz photonic devices and sensors.

## Data Availability

All data generated or analyzed during this study are included in this published article.

## Abbreviations

PIT: Plasmon-induced transparency
FDTD: Finite-difference time-domain
CMT: Coupled mode theory
SPPs: Surface plasmon polaritons
PIA: Plasmon-induced absorption
MDA: Modulation degree of amplitude
